# Untangling an insect’s virome from its endogenous viral elements

**DOI:** 10.1186/s12864-023-09737-z

**Published:** 2023-10-24

**Authors:** Paula Rozo-Lopez, William Brewer, Simon Käfer, McKayla M. Martin, Benjamin J. Parker

**Affiliations:** 1 Department of Microbiology, University of Tennessee, Knoxville, TN 37916 USA; 2 Institut Für Biologie Und Umweltwissenschaften, Carl Von Ossietzky Universität Oldenburg, 26129 Oldenburg, Germany

**Keywords:** Viral discovery, RNAseq, Insects, Aphids, Endogenous viral elements

## Abstract

**Background:**

Insects are an important reservoir of viral biodiversity, but the vast majority of viruses associated with insects have not been discovered. Recent studies have employed high-throughput RNA sequencing, which has led to rapid advances in our understanding of insect viral diversity. However, insect genomes frequently contain transcribed endogenous viral elements (EVEs) with significant homology to exogenous viruses, complicating the use of RNAseq for viral discovery.

**Methods:**

In this study, we used a multi-pronged sequencing approach to study the virome of an important agricultural pest and prolific vector of plant pathogens, the potato aphid *Macrosiphum euphorbiae*. We first used rRNA-depleted RNAseq to characterize the microbes found in individual insects. We then used PCR screening to measure the frequency of two heritable viruses in a local aphid population. Lastly, we generated a quality draft genome assembly for *M. euphorbiae* using Illumina-corrected Nanopore sequencing to identify transcriptionally active EVEs in the host genome.

**Results:**

We found reads from two insect-specific viruses (a *Flavivirus* and an *Ambidensovirus*) in our RNAseq data, as well as a parasitoid virus (*Bracovirus*), a plant pathogenic virus (*Tombusvirus*), and two phages (Acinetobacter and APSE). However, our genome assembly showed that part of the ‘virome’ of this insect can be attributed to EVEs in the host genome.

**Conclusion:**

Our work shows that EVEs have led to the misidentification of aphid viruses from RNAseq data, and we argue that this is a widespread challenge for the study of viral diversity in insects.

**Supplementary Information:**

The online version contains supplementary material available at 10.1186/s12864-023-09737-z.

## Introduction

The last decade has transformed our understanding of the viral communities associated with insects, the most abundant and diversified animal group [1–4]. Insect viruses have been primarily studied in the context of vector-borne pathogens, which are transmitted horizontally between insect vectors and amplifying hosts, and often have medical or agricultural relevance. Other viruses, however, only replicate within the insect and are maintained in natural populations through horizontal and/or vertical transmission. These insect-specific viruses have important impacts on host biology [5–7], but much work remains to be done to describe insect-specific viral diversity and to uncover the hidden role viruses play in insect phenotypes and evolution [8–10].

To address this gap, researchers have employed high-throughput approaches to viral discovery, including next-generation sequencing and analysis of RNA [2, 11–15]. However, there are several serious limitations to this approach. For example, RNAseq data does not distinguish between reads that come from viruses infecting insect cells or from microbes infecting an organism ingested by the insect. Another potential challenge with using RNAseq for viral discovery is that insects often harbor fragments of viral sequences in their genomes. The endogenous viral elements (EVEs) described to date have homology with multiple clades of single- and double-stranded DNA and RNA viral families [16]. We have a limited understanding of the role EVEs are playing in insect biology, but transcriptionally active EVEs have been shown to play functional roles in regulating host genome stability and as an antiviral defense against exogenous viruses [17–19]. EVEs are remarkably common across insects [20], and thus EVEs could represent a widespread challenge for the use of RNAseq in viral discovery.

Aphids (Hemiptera: Aphidoidea) are hosts to diverse viruses, including plant pathogens with agricultural significance and insect-specific viruses [21, 22]. Recent studies have used metatranscriptome sequencing to describe viral diversity in aphids [23–29], and have found insect-specific DNA viruses in the family *Parvoviridae* and RNA viruses in the *Bunyaviridae*, *Dicistroviridae*, *Flaviviriridae*, *Iflaviridae*, and *Mesoniviridae* families [21]. The potato aphid *Macrosiphum euphorbiae* (Thomas, 1878) is an important cosmopolitan agricultural pest that infests tomatoes, potatoes, and other economically important crops [30]. *M. euphorbiae* is also an important vector of plant viruses (Families *Bromoviridae*, *Closteroviridae*, *Geminiviridae*, *Potyviridae*, and *Solemoviridae*) and was recently shown to host several insect-specific viruses belonging to the families *Flaviviridae* (genus *Flavivirus*) and *Parvoviridae* (genus *Ambidensovirus*) [24, 31, 32]. Despite *M. euphorbiae's* economic importance, no genomic resources were available outside body and salivary gland transcriptomes [24, 33, 34].

The genomes of multiple aphid species have been shown to harbor EVEs that mediate growth, development, and wing plasticity [35–39]. In this study, we use next-generation sequencing and analysis to show that aphid EVEs have led to the misidentification of aphid viruses from RNAseq data. First, we used RNAseq to characterize the microbial diversity of field-collected *M. euphorbiae* adults, and found sequences from two insect-specific viruses that have been identified previously in aphids, a *Flavivirus* and *Ambidensovirus*. Then, we generated a high-quality draft genome sequence for this species. Our genome showed that insect-specific Ambidensoviral hits corresponded to transcriptionally active EVEs, indicating that a previously described virus is actually an endogenous viral element in the *M. euphorbiae* genome. These EVEs have homology to the ‘APNS’ genes in the related pea aphid (*Acyrthosiphon pisum*), which resulted from a lateral gene transfer from a *Densovirus* and play a role in the plastic production of aphid wings [37]. Our study illustrates how careful analysis using multiple methods is needed to untangle insect viromes from EVEs and furthers our understanding of the surprisingly widespread presence of Densoviral EVEs in aphid genomes.

## Methods

### Aphid collection

We collected asexual winged and wingless female *M. euphorbiae* adults from cultivated tomato plants (var Husky Cherry Red) in Knoxville, TN, USA, between April and June 2021 and 2022. We stored individual aphids in 1.5 mL Eppendorf tubes (Eppendorf, Hamburg, Germany) at -80 °C until processing. For species taxonomy validation, *M. euphorbiae* (NCBI TaxID: 13131), we used COI barcoding (LCO1490 5'-GGTCAACAAATCATAAAGATATTGG-3' and HCO2198 5'-TAAACTTCAGGGTGACCAAAAAATCA-3'), sanger sequencing, and comparisons of our COI sequences to the Barcode of Life Data System (https://www.boldsystems.org/) [40]. Our COI barcode sequence was uploaded to NCBI with accession number OQ588703. All *M. euphorbiae* samples were labeled as “Me” followed by a consecutive number.

### Cultivation of *M. euphorbiae* strain Me57

To establish a colony of *M. euphorbiae* in the laboratory, we used a single asexual female collected in 2021. After colonization, we maintained this line on tomato plants (Husky Cherry Red) at 20 °C 16L:8D. We screened the line for the seven species of facultative symbionts found in aphids using established PCR protocols [41, 42]. For this screen, we extracted DNA using ‘Bender buffer’ and ethanol precipitation as in previous studies [43, 44]. We then used PCR with species-specific primers [42, 45] to screen for *Hamiltonella defensa, Fukatsuia symbiotica* (previously referred to as X-type), *Regiella insecticola*, *Rickettsia* sp., *Ricketsiella* sp., *Serratia symbiotica*, and *Spiroplasma* sp. following the recommended thermal protocol (94 °C for 2 min, 11 cycles of 94 °C for 20 s, 56 °C (declining 1 °C each cycle) for 50 s, 72 °C for 30 s, 25 cycles of 94 °C for 2 min, 45 °C for 50 s, 72 °C for 2 min, and a final extension of 72 °C for 5 min).

### RNA extraction and sequencing

We randomly selected four *M. euphorbiae* samples (Me022, Me112, Me152, Me202) for further analysis. We homogenized single aphids with a pestle in 500 µL of TRIzol (Invitrogen; Thermo Fisher Scientific, Inc., Waltham, MA, USA) to extract total RNA using BCP (1-bromo-3-chloropropane; Life Technologies, Thermo Fisher Scientific, Inc., Waltham, MA, USA) with isopropanol precipitation. We used the Zymo RNA Clean & Concentrator kit (Zymo Genetics Inc., Seattle, WA, USA) to improve the purity and to remove gDNA using DNAse I. We then performed metatranscriptome sequencing at Novogene (Novogene Corporation Inc., Sacramento, CA, USA). Library preparation was conducted using ribosomal RNA (rRNA) depletion by Illumina TruSeq Stranded Total RNA with Ribo-Zero Plus and NEBNext rRNA Depletion Kit (Zymo Genetics, Inc., Seattle, WA, USA). The libraries were sequenced to approximately 9 billion base pairs (bp) per sample with 150 bp paired-end reads on an Illumina NovaSeq platform. Raw reads were deposited into the NCBI Sequence Read Archive under BioProject ID PRJNA942253 with BioSample accessions SAMN33770905-SAMN33770908, and data accessions SRR23870213-SRR23870216.

### Microbial analysis using CZ ID

We assessed the success of ribosomal reduction in the metatranscriptome libraries using riboPicker [46] and the reference database SILVA_138 [47] (S1 Table). We then used the CZ ID platform pipeline V7.1 (https://czid.org) [48], a cloud-based, open-source bioinformatics platform designed to detect microbes from metagenomic data. We removed host-specific reads (STAR host subtraction) using the *Acyrthosiphon pisum* genome [49], trimmed adapters using Trimmomatic [50], removed low-quality reads with PriceSeqFilter [51], and aligned the remaining reads to the NCBI NT and NR databases using Minimap2 [52] and Diamond [53]. In parallel, short reads were de novo assembled using SPADES [54] and mapped back to the resulting contigs using bowtie2 [55] to identify the contig to which each raw read belongs. We used the CZ ID water background model, which evaluates the significance (z-scores) of relative abundance estimates for microbial taxa in each sample. Potential bacterial reads were distinguished from contaminating environmental sequences by establishing z-score metrics ≥ 10, alignment length over 50 matching nucleotides (NT L ≥ 50), and a minimum of five reads per million aligning to the reference protein database (NR rPM ≥ 5). Potential viruses were established by z-score metrics of ≥ 1, NT L ≥ 50, NR rPM ≥ 1, and a minimum of five reads per million aligning to the reference nucleotide database (NT rPM ≥ 5) [48, 56, 57]. Bacterial and viral contigs were confirmed with BLASTX and BLASTN manual searches. Only annotated microbial hits with revised taxonomy through manual BLAST searches were used for further analysis. The “Macrosiphum euphorbiae” project is publicly available via CZ ID.

### Analysis of the *Flavivirus* MeV-1 genome

We used the CZ ID viral consensus genomes pipeline to build a consensus genome from the sample with *Macrosiphum euphorbiae virus 1* (MeV-1) present at high levels (Me202). In short, contigs were aligned to the reference MeV-1 genome (NCBI Entry KT309079.1) using minimap2 [52] and then trimmed using TrimGalore (Phred score < 20) [58]. The consensus genome was generated with iVar consensus using a depth of five or more reads [59]. Our consensus genome was deposited into the NCBI with accession number: OQ504571.

### Analysis of the *Ambidensovirus* using de novo assembly and TRAVIS

We conducted an additional screening and viral genome assembly of potential Ambidensoviruses using de novo transcriptome assemblies as follows. We used Trimmomatic v.0.39 [50] to trim the sequence adapters and filtered low-quality/complexity reads, and we assessed for post-trimming quality using FastQC v.0.11.9 [60]. Then, we used Trinity v.2.14 [61] to de novo assemble the remaining reads. We used TRAVIS (v.20221029, https://github.com/kaefers/travis) to scan the assembled transcriptomes for *Densovirus*-like sequences. We built the reference database to the *Parvoviridae* viral family including the accepted *Densovirinae* viral species by the International Committee on Taxonomy of Viruses (ICTV) by 29^th^ Oct 2022 (S2 Table), extracted open reading frames between 100 and 2000 amino acids from the assembled transcriptomes, and screened using HMMER v3.3.1 [62], MMSeqs2 [63], BLASTP v2.12.0 [64], and Diamond v2.0.15 [53]. We set the e-value cutoff at 1 × 10^–6^, where applicable. All hits were again searched with Diamond against the non-redundant protein database (NCBI, downloaded on 29 Oct 2022).

### MeV-1, MeV-2, and *Hamiltonelladefensa* screening

Like all aphids, *M. euphorbiae* hosts an obligate heritable bacterial symbiont called *Buchnera aphidicola* that synthesizes amino acids missing from the aphid’s diet of plant phloem, and can also harbor several other facultative symbiotic bacteria (listed above) [45]. To screen for these microbes, we used 1 μg of total RNA extracted (as above) from each of the 23 adults collected during 2022 for cDNA synthesis with iScript cDNA synthesis kit, which uses random hexamer primers (Bio-Rad Laboratories, Inc., Hercules, CA, USA). To screen for the *Flavivirus, Macrosiphum euphorbiae virus 1* (MeV-1), we used 100 ng of cDNA, the primers MevirF1 (5'-GTACACTTGCCTTACCTTACTGT-3') and MevirR1b (5'-AACACGGGTCACGACCTTAG-3'), and the PCR conditions previously described [32]. To screen for the *Ambidensovirus, Macrosiphum euphorbiae virus 2* (MeV-2), we used 100 ng of cDNA, the MeV2-F (5'-CCGGATGACAAATCCCACGA-3') and MeV2-R (5'-AATAGGCGCAGAGATGGACG-3') primers, and the recommended PCR conditions [24]. In addition, we extracted DNA from our laboratory aphid colony Me57 (as above) and used 40 ng of genomic DNA to screen for MeV-2. The aphid Glyceraldehyde 3-phosphate dehydrogenase (G3PDH) was used as internal control (primers G3PDH_F (5'-CGGGAATTTCATTGAACGAC-3') and G3PDH_R (5'- TCCACAACACGGTTGGAGTA-3') [37]). Moreover, we used 200 ng of the cDNA previously synthesized for MeV-1 and MeV-2 screening and the protocols for *Hamiltonella defensa* PCR screening (as described above) to evaluate the proportion of field-collected aphids harboring this bacterial symbiont (S3 Table). We used a non-parametric (Spearman) correlation to investigate the potential interaction between *Hamiltonella* and MeV-1.

### DNA extraction and sequencing

We pooled seven genetically identical adult unwinged aphids from our Me57 laboratory line and isolated genomic DNA (gDNA) using a phenol/chloroform extraction. We then sheared the gDNA to approximately 20 kb fragments using Covaris G-tubes (Covaris LLC., Woburn, MA, USA) at 4,200 RMP for 1 min, followed by tube inversion. For library preparation, we used the NEB Next PPFE repair kit with Ultra II end prep reaction (New England Biolabs, Ipswich, MA, USA) under the recommended conditions and Nanopore ligation sequencing kit SQK-LSK110. For sequencing, we used a Nanopore R9.4.1 (FLO-MIN106D) flow cell and a MinION MIN-101B sequencing device (Oxford Nanopore Technologies, Oxford, UK). We ran the flow cell for 24 h, followed by a wash with Flow Cell Wash Kit (EXP-WSH004); we then reloaded the flow cell with a second library prep and ran the sequencer for an additional 48 h. We stopped the second sequencing run at 72 h (~ 22 Gbps of sequencing). In addition, we performed an additional 5.3 Gb of 150 bp paired-end sequencing to polish the assembly on an Illumina NovaSeq platform. DNA was extracted as above, and library prep and sequencing were performed by Novogene Inc. Raw reads were filtered for low quality and adapter contamination by Novogene Inc.

### *M. euphorbiae* whole genome assembly

We used Guppy (Oxford Nanopore Technologies) for base-calling and quality trimming raw reads. For the removal of *Buchnera* reads, we used minimap2 v.2.24 [52] in conjunction with SAMtools v.1.15.1 [65] to map our reads against the *Buchnera aphidicola* (strain *Macrosiphum euphorbiae*) genome (NCBI accession NZ_CP029205) and the corresponding plasmids (NCBI accession number NZ_CP029203 and NZ_CP029204). We only kept unmapped reads for aphid genome assembly. We assembled Nanopore reads using CANU v.2.0 [66] with an estimated genome size of 541 Mbp. We removed allelic variants from the assembly using purge_haplotigs v.1.1.2 [67], first by mapping reads to the assembly using minimap2 v2.24-r1122 with Samtools v.1.15.1 and manually choosing cutoffs for haploid vs. diploid coverage based on a histogram plot (v -l 5 -m 27 -h 60), and then by purging duplicated contigs based on coverage level (-j 80 -s 50). For assembly polishing, we used the Illumina reads after quality assessment using FastQC V0.11.9 [60]. Then we used these reads to polish the purged assembly using Pilon v.1.24 with default parameters [68]. We used BlobTools2 [69] to identify remaining contaminating contigs. For this, we used blast results obtained from the BLASTN function against the NT database using blast plus v.2.12.0 [70], read coverage obtained by mapping the Illumina reads to the assembly using minimap2 v.2.24 [52], and GC content in this analysis. Based on these results, we removed all the short contigs with strong homology to the plant genus *Solanum* (which includes the tomato host plant species of *M. euphorbiae*) as we suspect these contigs were assembled from host plant contamination in the guts of sequenced aphids. We also removed two short contigs with homology to other bacterial contaminants such as *Escherichia coli* and *Pseudomonas* sp. We removed a contig nearly identical to the pLeu plasmid found in *Buchnera aphidicola*. We also removed small portions of two larger contigs, which matched the *Buchnera* genome and had been misassembled into the larger contigs. The final annotation was assessed using BUSCO v.5.3.2 [71] with the MetaEuk gene predictor [72] implemented in galaxy.org, using the hemiptera_odb10 (2020–08-05) lineage dataset. This Whole Genome Shotgun project has been deposited at DDBJ/ENA/GenBank under the accession JARHUA000000000. The version described in this paper is version JARHUA010000000.The raw Nanopore (SRR23851809) and Illumina reads (SRR23919025) associated with the genome are available through the Sequence Read Archive, and the finished assembly is available with accession number SRR23851809.

### Characterizing endogenous viral elements in the *M. euphorbiae* genome

DNA Illumina raw reads were used as input to the CZ ID platform pipeline V7.1 (https://czid.org) and a z-score metrics of ≥ 1 and NT L ≥ 50 as described above [48, 56]. Additionally, to screen for actively transcribed *Densovirus*-like EVEs in the *M. euphorbiae* genome, we used BLASTN searches using the seven viral hits provided as individual Trinity contigs flagged by TRAVIS (sequences available as S1 file) against the genome scaffolds. All non-redundant hits from these searches with e-values < 1.10^–3^ were extracted and used in further analyses [35].

## Results

### Analysis of non-host sequences detected in single aphids

**Fig. 1:**
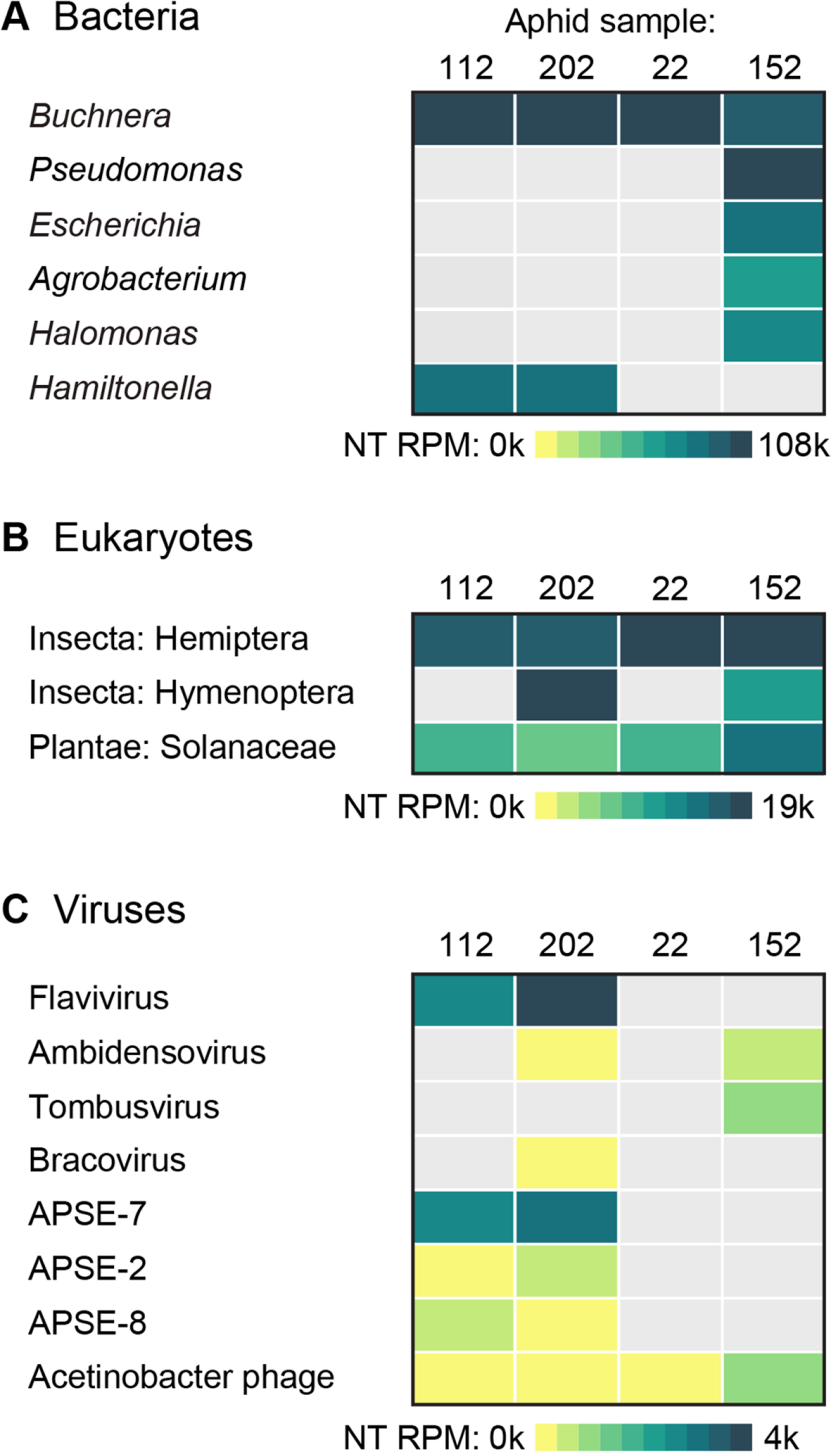
Details of the per sample breakdown of reads aligning to specific bacterial (**A**), eukaryotic (**B**), and viral (**C**) taxa. Reads per million aligned to the nucleotide database (NT rPM) was used as the quantitative metric in the heatmaps (see Table S5 for metric details)

We used the pea aphid (*A. pisum*) genome to subtract host reads from our transcriptome data set. On average, 81.8% of the reads mapped to *A. pisum* and were removed from further analysis (S1 Table). For each sample, we then analyzed the remaining reads as the overall proportion of assembled reads assigned to bacterial, eukaryotic, and viral taxa (public project “Macrosiphum euphorbiae” at https://czid.org). Bacterial taxa dominated the microbial signature (Fig. 1A), and as expected, the highest number of hits matched the aphid obligate symbiont *Buchnera aphidicola* with over 45,000 reads per million aligning to the nucleotide database (NT rPM > 45,000). Hits to an aphid facultative symbiont (*Hamiltonella defensa)* were found in two samples (NT rPM > 8,700). Moreover, one sample (Me152) showed a strong signature of bacterial contaminants (*E. coli, Pseudomonas, Halomonas,* and *Agrobacterium*) that are commonly present in soil and plant surfaces.

In terms of eukaryotes (Fig. 1B), we found hits to Solanaceae, which includes the host plant species of *M. euphorbiae,* and Brachonidae parasitoid wasps (Insecta: Hymenoptera) in two samples (NT rPM > 18,000). *M. euphorbiae* is known to be parasitized by hymenopterous wasps belonging to the superfamilies Ichneumonoidea (Braconidae) and Chalcidoidea [73]. In addition, there were some *M. euphorbiae* species-specific reads remaining, which did not map to the pea aphid reference genome but showed some homology to other aphid species (Insecta: Hemiptera).

Regarding the virome, we detected the presence of two insect-specific viruses in our metatranscriptome data (Fig. 1C). The highest number of hits matched a previously described insect-specific *Flavivirus*, called *Macrosiphum euphorbiae virus 1* (MeV-1) [32], which was detected in two samples (NT rPM = 234 and 4055 for Me112 and Me202, respectively). We also detected viral hits to an insect-specific *Ambidensovirus* (Me202 and Me152; NT rPM 1.3 and 1.8, respectively). Other viral reads in our samples included a *Bracovirus* in one of the samples that was parasitized with the Brachonidae wasp (Me202; NT rPM = 1) and a *Tombusvirus* (Me152; NT rPM = 2.9), a family of plant pathogenic viruses with a single-stranded positive-sense RNA genome. Lastly, we detected two phage genera, the *Hamitonella-*specific phage A. pisum secondary endosymbiont (APSE; NT rPM > 310), in the same samples found positive for this symbiont (Me112 and Me202). We also found Acinetobacter phage (NT rPM 0.5–18) in all samples, which is a bacteriophage highly prevalent in the environment [74].

### Analysis of insect-specific viruses

**Fig. 2:**
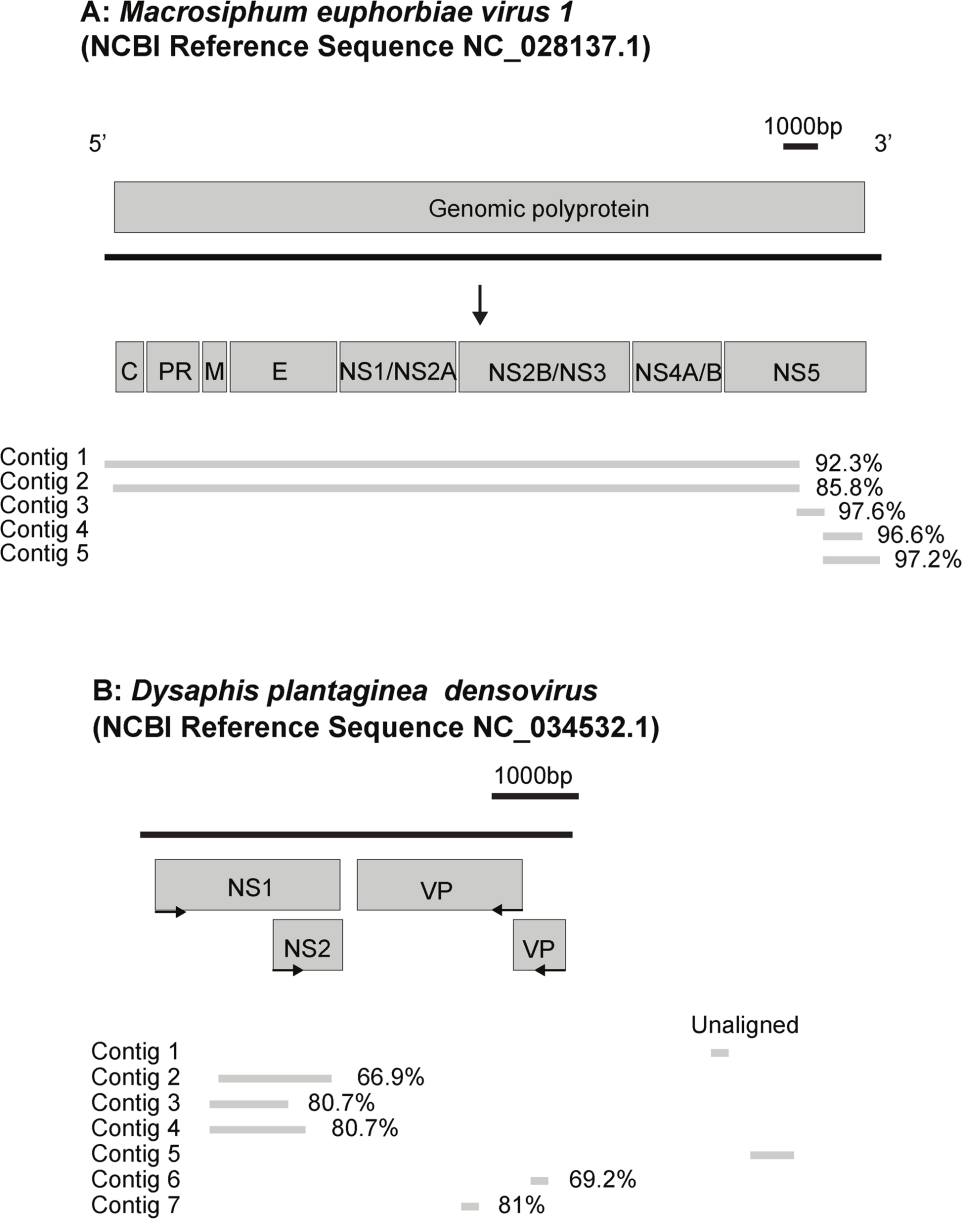
Assembled *M. euphorbiae* transcriptome contigs aligning to previously a described insect *Flavivirus* (**A**) and an *Ambidensovirus* (**B**)

Five assembled contigs aligned to the MeV-1 reference genome (NCBI accession KT309079) with nucleotide identity ranging between 85.8–97.2% (Fig. 2A). Transcriptome data from our field samples retrieved 17,397 informative nucleotides allowing the assembly of a nearly complete genome for MeV-1. Our MeV-1 consensus genome has a coverage breadth of 79% and a coverage depth of 673.2x (NCBI accession OQ504571) (S1 Figure). This single-stranded positive-sense RNA genome contains a single large ORF encoding a polyprotein of 7,333 amino acids, which is subsequently processed to generate structural and non-structural proteins [75]. Previous analysis indicated that the polyprotein motifs of MeV-1 helicase, methyltransferase, and RNA-dependent RNA polymerase (RdRp) are similar to domains in other *Flaviviruses* (family *Flaviviridae*) [21, 32]. The characteristic secondary structures (RNA stem-loop) in *Flavivirus* genomes most likely contributed to the 5,283 missing bases in our MeV-1 consensus genome assembly [76].

In addition, we detected two contigs with 80% nucleotide similarity to the non-structural protein 1 (NS1) of *Dysaphis plantaginea densovirus* (DplDNV), a single-stranded DNA insect-specific *Ambidensovirus* (family *Parvoviridae*) (S2 File). Due to the lack of a publicly available genome or partial viral sequences of *Macrosiphum euphorbiae virus 2* (MeV-2), an *Ambidensovirus* previously described in the same aphid species [24], we were not able to explore the homology between both viruses. Therefore, we conducted a more extensive analysis of our RNAseq data using TRAVIS, a consistency-based virus detection pipeline for sensitive mass screening of transcriptomic data directed toward *Parvoviridae* proteins. In general, sequence identity between *Densovirinae* (a subfamily of viral species exclusively infecting arthropods) is very low, with some pairs sharing < 15% amino acid identity some of their viral proteins. However, *Densoviruses* often express conserved domains in the NS1 and VP proteins, which are useful for phylogenetic inferences [77]. We found seven *Densovirus*-like hits (S1 File) and used them to construct a hypothetical genome assembly using DplDNV (NCBI accession NC034532) as a reference (Fig. 2B). We found three contigs with 68.8% to 81.3% nucleotide similarity to the non-structural ORF1 (encoding for the NS1 protein) and two contigs with 68.8% to 86.2% nucleotide similarity to the structural ORF (encoding for the VP protein). None of the assembled contigs had either nucleotide or amino acid similarity to DplDNV ORF2 (encoding for the NS2 protein). Importantly, all densoviral NS1-like sequences also had 72% to 85% nucleotide similarity to the pea aphid APNS-2 (NCBI accession NC042493.1 and NC042494.1), an endogenous viral element (EVE) that contributes to wing phenotypic plasticity in this species [37].

### Insect-specific virus frequency in natural populations

**Fig. 3:**
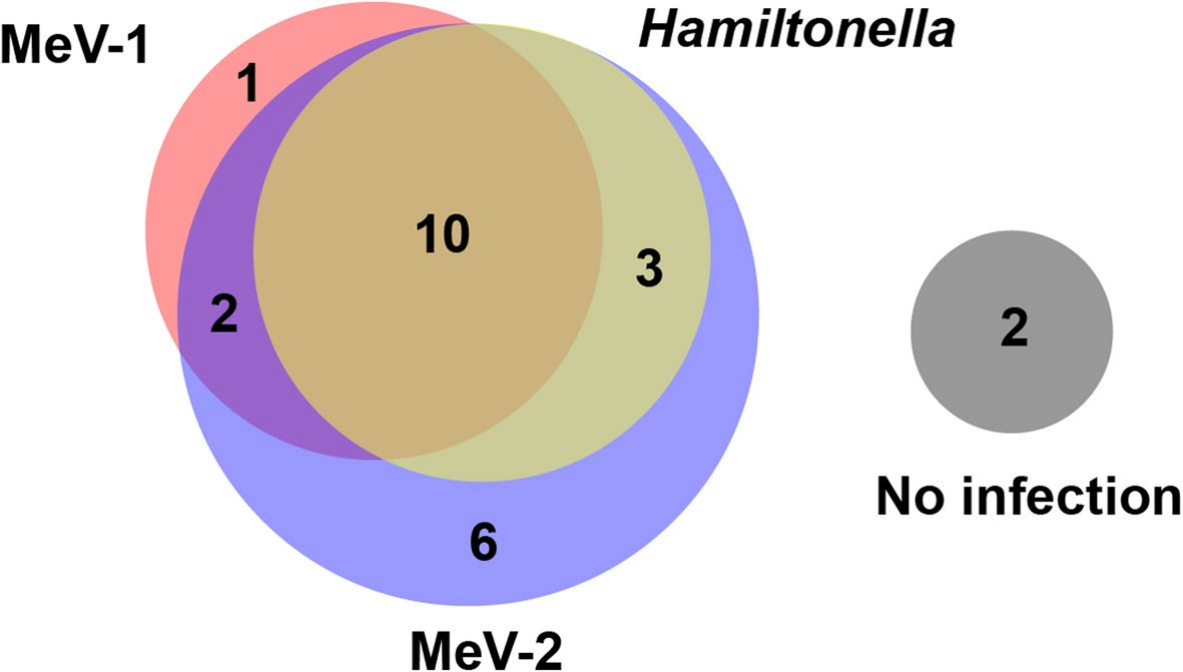
Frequency of *Macrosiphum euphorbiae virus 1* (MeV-1), *Macrosiphum euphorbiae virus 2* (MeV-2), and *Hamiltonella denfesa* infections in wild-collected (*n* = 23) and Me57 laboratory established (*n* = 1) aphids. All samples were tested using cDNA from individual aphids for PCR screenings

To further investigate the infection frequency of MeV-1 and MeV-2 infections in natural populations, we used a PCR approach to screen 23 individual adult aphids collected in 2022 as well as aphids from our colonized *Macrosiphum* line (Me57). We found that only 13 field-collected aphids were positive for MeV-1 (54.2%) and 21 aphids (87.5%) were positive for MeV-2, including the laboratory established line (Me57) (Fig. 3). We also tested the cDNA of field-collected aphids (previously screened for MeV-1) for the presence of *Hamiltonella defensa* and found that 54.2% of the aphids (*n* = 13) were harboring this bacterial symbiont. We found that 41.7% of individuals (*n* = 10) tested positive for both the *Flavivirus* and *Hamiltonella* (Fig. 3), but this association was not statistically significant (*p*-value = 0.078; *r* = 0.375).

### Genome sequencing for analysis of endogenous viral elements (EVEs)

Since our laboratory line (Me57) was found to be PCR positive for MeV-2, we used the CZ ID platform to identify viral taxa using the Illumina DNA reads from our colonized Me57 aphid line. Surprisingly, we detected only a single contig with a low number of Ambidensoviral hits (NT rPM > 0.329), which also showed 79.0% nucleotide similarity to the DplDNV NS1 and 84.34% nucleotide similarity to an uncharacterized genomic transcript in pea aphids (NCBI accession XM_029492170.1). Since both our transcriptomic (Fig. 2B) and genomic data were unable to recover a complete or near-to-complete *Ambidensovirus* genome, we suspected that these viral reads could correspond instead to actively transcribed EVEs, as previously reported in other closely related aphid species [35, 37].

**Fig. 4:**
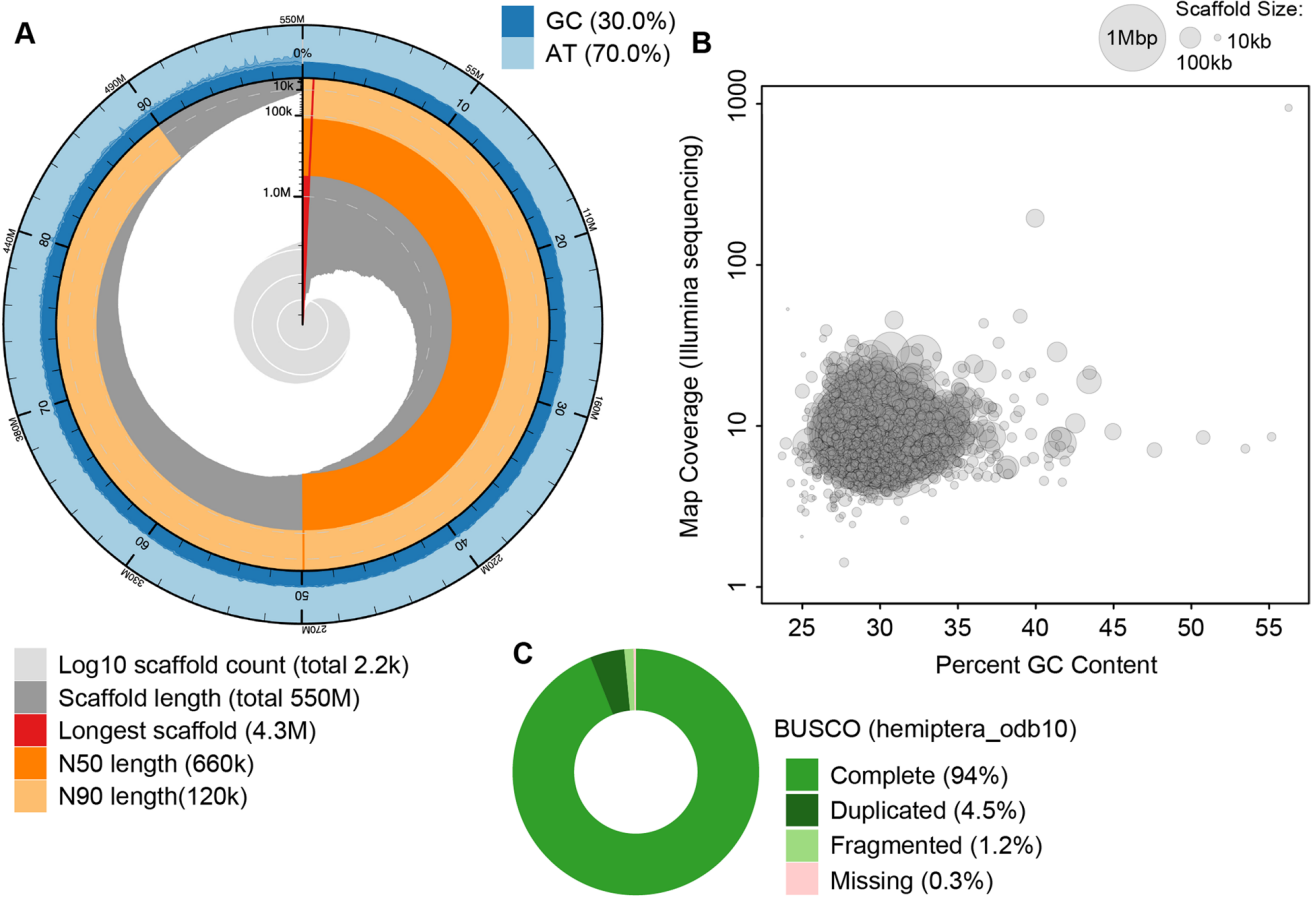
*M. euphorbiae* genome assembly metrics (**A**), GC content and coverage (**B**), and BUSCO metrics (**C**)

To determine with certainty whether the Ambidensoviral hits found in our transcriptome data corresponded to an actively transcribed EVE, we assembled the first *M. euphorbiae* genome publicly available. We obtained a total of 4,223,264 nanopore reads (at an average of 5.21 kb) and 35,578,886 Illumina reads (PE 150 bp) from sequencing. After assembly, haplotig purging, polishing, and manual removal of plant and bacterial contigs, our assembly contained 2,176 contigs with an N50 length of 665 kb and a total length of 545.7 Mb (Fig. 4A). *M. euphorbiae* has a similar GC content (29.96%; Fig. 4B) to other sequenced aphids (e.g., *Acyrthosiphum pisum* at 29.6%, *Myzus persicae* at 30.1%, and *Aphis glycines* at 27.8%) [78, 79]. The size of our assembly is close to a recent estimation of the *M. euphorbiae* genome size based on flow cytometry which was estimated at 531.7 Mb [78]. Similarly, an analysis of single-copy orthologs showed our assembly contains 98.5% complete BUSCOs, with 94% present in single copies and 4.5% duplicated (Fig. 4C). An additional 1.2% of BUSCOs are fragmented, and 0.3% are missing. Together these results suggest that this draft of the genome is highly complete.

We then used this genome as a reference to screen for the seven individual Trinity contigs flagged by TRAVIS as potential *Ambidensovirus* in our previous analysis (S1 File). Initially, we selected hits with e-values < 1.10^–3^ [35]; however, most of the 3,044 hits represent shorter sequences rather than the actual transcript length (S4 Table); therefore, we restricted the search to matches consistently to the entire length of each transcript and e-values = 0 (Table 1). No full-length hits in the genome were found for the two largest viral contigs (contig3 and contig4); instead, the best hits for these two contigs corresponded to 16–17% of the total length. In insects, the EVE repertoires vary between distinct populations of a given species and, in some cases, even between individuals within the same population [80]. This phenomenon potentially explains why all the field aphid samples (*n* = 3) that tested negative for MeV-2 by PCR amplified a product of approximately 500 bp, which is about half of the expected size reported for the primers used. Given that the genome assemblies and RNAseq data sets were derived from different aphid strains, it is not surprising the wide range of partial-length Ambidensoviral hits obtained in our analysis. However, we are confident that five full-length viral transcripts are constitutively expressed from three regions of the *M. euphorbiae* genome (tig00030708_pilon, tig00029914_pilon, and tig00027226_pilon).

**Table 1:** List of Ambidensoviral transcripts and the corresponding integrations in *M. euphorbiae* genome

Transcriptome contig	Trans-cript length	Percent-age of identical sites	Hit end	Hit start	Genome contig	Query end	Query start
Travis_contig1	783	96.70%	783	1	tig00030708_pilon	198038	197258
Travis_contig1	783	98.90%	1	783	tig00029914_pilon	60345	59559
Travis_contig2	466	96.20%	416	1	tig00030708_pilon	198433	198018
Travis_contig2	466	99.80%	1	466	tig00029914_pilon	59579	59114
Travis_contig3	2155	-	-	-	-	-	-
Travis_contig4	2878	-	-	-	-	-	-
Travis_contig5	1174	99.90%	1	1174	tig00030708_pilon	92758	91585
Travis_contig6	1040	99.80%	1	1040	tig00030708_pilon	85562	84525
Travis_contig7	635	100.00%	635	1	tig00030708_pilon	86191	85557
Travis_contig7	635	84.90%	635	1	tig00027226_pilon	138266	137632

## Discussion

RNAseq is becoming an essential tool for virus discovery. Our study illustrates how endogenous viral elements in host genomes can be an obstacle to using RNAseq for characterizing viral diversity in arthropods. We used rRNA-depleted RNAseq along with bioinformatic tools to characterize the virome of an important insect pest species, the potato aphid *Macrosiphum euphorbiae.* Our analysis found sequences from two insect-specific viruses from the genus *Ambidensovirus* and *Flavivirus* described in previous RNAseq studies [24, 32]. However, only by sequencing and assembling the genome of this insect were we able to demonstrate that the previously described *Ambidensovirus* is a transcriptionally active EVE rather than an exogenous virus. Endogenous viral elements are abundant in arthropod genomes, and thus our study illustrates how EVE sequences in RNAseq studies are an important consideration for future studies of viral diversity in arthropods.

The EVEs we describe in *M. euphorbiae* have significant homology with those recently described in the pea aphid*.* It was recently shown that two copies of a transcribed densoviral non-structural protein 1 (termed the “*A. pisum* non-structural” or “APNS” genes) are upregulated in response to crowded conditions and are functionally linked to the plastic production of wings [37]. These genes originated from a lateral gene transfer from *Dysaphis plantaginea densovirus* (DplDNV), which, when infecting rosy apple aphids, causes their host to develop wings in greater proportion than non-infected aphids [81]. It appears that the function of these viral genes had been conserved after endogenization but additional data is needed to decipher the role they these EVEs are playing in *M. euphorbiae*. Together with recent findings, our data show that the APNS genes are widespread through the tribe Macrosiphini [35, 37, 39, 82], raising interesting questions about the origins of these EVEs in this phylogenetic group and their role in host biology.

Endogenization of *Parvoviruses* (including *Ambidensovirus*) may be favored by the double-stranded DNA intermediate that occurs during nuclear replication, the endonuclease activity of NS1 protein, and the eukaryote double-stranded break repair mechanism [83, 84]. Previous studies have estimated that around 10% of the parvoviral sequences described in animals are likely integrated into host genomes [77]. In most cases, the EVE status of *Parvovirus*-like sequences remain uncertain due to unavailable or incomplete genomes for those species in which transcriptome data is available [77]. Importantly, multiple recent studies have used RNAseq data to describe the presence of aphid-specific Densoviruses [23, 85]. These studies often rely only on partial sequences of the one viral protein that is most susceptible to endogenization (NS1). As demonstrated by our results, NS1 viral transcripts do not always indicate that the reported high frequency infections are produced by an exogenous *Ambidensovirus*, and these results should be interpreted with caution in future studies.

Lastly, our study sheds light on the biology of MeV-1, an insect-specific *Flavivirus* (family *Flaviviridae*), previously characterized by RNAseq studies along with replication intermediaries (dsRNA) of *M. euphorbiae* populations collected in France [32]. We found that this virus, contrary to previous reports, is present in a North American population of *M. euphorbiae*, and we found that it is highly prevalent in our samples. By assembling a near-to-complete genome of MeV-1 from our RNAseq data and following the criterion to define *Flavivirus* species via nucleotide sequence comparisons [86], we consider that our local aphid population is infected with the same viral species (as it shared over 84% pairwise nucleotide homologies with the reference virus) but a distinct viral strain (4% nucleotide sequence difference). No obvious infection symptoms or abnormal phenotypes were observed in MeV-1 positive aphids. Future studies are needed to determine what phenotypic effects this virus has on its host.

Since EVEs are common in insect genomes [18], our results highlight a widespread challenge in studying insect viromes from RNAseq data. In future studies, it will be important to combine sequencing methodologies along with careful consideration of the biological characteristics and genome structure of putative novel viruses discovered. In aphids and other widely studied insects, the development of cultured cell lines is essential to isolate viral species described by sequence-based methods, to characterize viral replication, and to perform large-scale virus production that will facilitate future investigation of the complex interactions between insect-specific viruses and their hosts [21].

## Conclusions

We show that aphid EVEs have led to the misidentification of aphid viruses from RNAseq data. EVEs are common in insect genomes, and our results highlight a widespread challenge in studying insect viromes. We suggest that combining sequencing methodologies (e.g., RNA and whole genome sequencing) is necessary to overcome the potential pitfalls of RNAseq-based viral discovery.

### Supplementary Information

**Additional file 1: S1 Table.** RNAseq reads report.

**Additional file 2: S2 Table.** Parvoviridae reference database.

**Additional file 3: S3 Table.** Metadata of the field-collected aphids screened for the Hamilltonella defensa bacterial symbiont.

**Additional file 4: S4 Table.** Expressed Ambidensoviral EVEs from M. euforbiae genome (SRR23851809).

**Additional file 5: S5 Table.** Quantitative metric in the heatmaps (data showed as reads per million aligning to the nucleotide database (NT rPM).

**Additional file 6: S1 Figure.** MeV-1 consensus genome coverage breadth and depth in comparison to the reference genome (KT309079.1).

**Additional file 7: S1 File.** Sequences of Trinity contigs flagged as Ambidensovirus sequences by TRAVIS.

**Additional file 8: S2 File.** Sequences of contigs flagged as Flavivirus and Ambidensovirus sequences by CZID.

## Data Availability

Species barcode sequence is available through NCBI accession OQ588703. Sequencing raw reads and genome assembly are available through NCBI BioProject PRJNA942253. Microbial analysis data is available through the CZID Macrosiphum euphorbiae project. MeV-1 genome through NCBI accession OQ504571. Additional sequence data of the viral contigs are included as Supplemental S1 File and S2 File.

## Abbreviations

DplDNV: *Dysaphis plantaginea densovirus*
EVEs: Transcribed endogenous viral elements
gDNA: Genomic DNA
G3PDH: Glyceraldehyde 3-phosphate dehydrogenase
MeV-1: *Macrosiphum euphorbiae virus 1*
MeV-2: *Macrosiphum euphorbiae virus 2*
NS1: Viral non-structural protein 1
ORF: Open reading frame
RNAseq: High-throughput RNA sequencing
rPM: Reads per million
rRNA: Ribosomal RNA
VP: Viral protein
