# Supplementary figures and images for: Untangling an insect’s virome from its endogenous viral elements
Source: BMC Genomics. 2023 Oct 24;24:636. doi: 10.1186/s12864-023-09737-z (PMC10594914; doi:10.1186/s12864-023-09737-z)

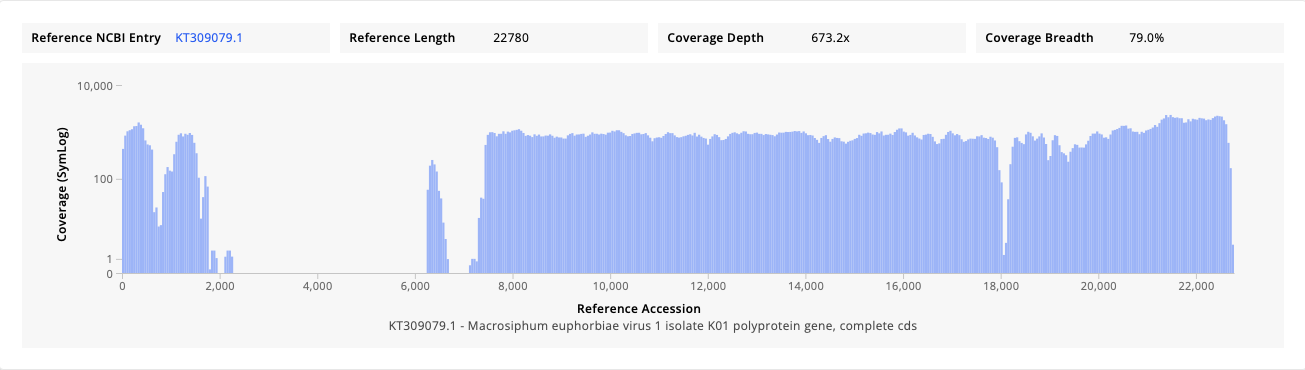

Supplement: Supplementary file 6 — Additional file 6: S1 Figure. MeV-1 consensus genome coverage breadth and depth in comparison to the reference genome (KT309079.1). [file 12864_2023_9737_MOESM6_ESM.tif]
